# Bilateral Anteriorly Displaced Microspherophakia in a Female Child With Marfanoid Habitus

**DOI:** 10.7759/cureus.38371

**Published:** 2023-05-01

**Authors:** Taimoor A Khan, Ali A Khan, Asfandyar Khan, Muhammad A Zahid, Mohammad A Mehboob

**Affiliations:** 1 Ophthalmology, National University of Medical Sciences (NUMS) Rawalpindi, Rawalpindi, PAK; 2 Ophthalmology, Armed Forces Institute of Ophthalmology, Rawalpindi, PAK; 3 Ophthalmology, Ayub Medical College, Abbottabad, PAK; 4 Ophthalmology, Monash Health, Clayton, AUS

**Keywords:** - marfan syndrome, marfan disease, alport’s syndrome, marfanoid habitus, microspherophakia, ectopia lentis

## Abstract

Microspherophakia is a rare congenital anomaly characterized by an abnormally small and spherical crystalline lens, which can be associated with several systemic syndromes. We present an extremely rare case of bilateral anteriorly displaced microspherophakia in a female child with Marfanoid habitus. The patient displayed phenotypic features resembling Marfan syndrome, including tall stature, muscle hypotonia, dolichostenomelia, and increased arm span than body length. However, unlike Marfan syndrome, Marfanoid habitus is not associated with mutations in the fibrillin-1 gene. The association between microspherophakia and Marfanoid habitus is a unique presentation that has not been reported in the literature. This case report aims to increase awareness of microspherophakia as a possible ocular association of Marfanoid habitus.

## Introduction

Microspherophakia is an uncommon congenital condition with a spherical lens and large anteroposterior length, and a smaller equatorial diameter. During the fifth and sixth months of embryogenesis, the nutritional deficiency of the tunica vasculosa lentis leads to the developmental arrest of secondary lens fibers. As an outcome, the preexisting zonular fibers weaken and lose tension, leading to the formation of a crystalline lens devoid of corticonuclear separation, and the lens is small and spherical [1]. Complications linked with microspherophakia include dislocation of the lens, subluxation of the lens, secondary glaucoma, and high lenticular myopia [2,3]. Due to the subsequent relaxation of the zonular fibers, the lens position is unstable, frequently resulting in the subluxation of the lens or its dislocation [4]. The small lens is frequently shifted towards the anterior chamber, which results in corneal endothelial cell loss and malfunction [5]. Microspherophakia may be isolated with complications such as angle closure glaucoma [6], but it can be present with other systemic conditions as well, such as Weill-Marchesani syndrome, Alport syndrome, hyperlysinaemia, Lowe’s syndrome, Peter’s anomaly, Marfan syndrome, homocystinuria, and Klinefelter syndrome [5]. Microspherophakia tends to run in families as well, where it can be genetically autosomal dominant or autosomal recessive [7]. However, microspherophakia has scarcely even been identified with Marfanoid habitus. Marfanoid habitus is an extremely rare congenital syndrome characterized by intellectual disability, flat face, and features resembling Marfan syndrome, including tall stature, muscle hypotonia, dolichostenomelia, and increased arm span compared to body length [8]. Marfanoid habitus is a clinical presentation with phenotypic features resembling Marfan syndrome. However, unlike Marfan syndrome, it is not related to any mutations in the gene for fibrillin-1, which encodes the glycoprotein that forms microfibrils in connective tissues. The genetic basis of Marfanoid habitus remains poorly understood, and it is unclear whether it represents a distinct clinical entity or a variant of Marfan syndrome [9]. The association between Marfanoid habitus and microspherophakia observed in our patient adds to the complexity of understanding the genetic and phenotypic heterogeneity of these conditions. We present an extremely rare case of bilateral microspherophakia with an anteriorly displaced lens in a female child with Marfanoid habitus. Our article discusses the rarity of this presentation as a manifestation of Marfanoid habitus and aims to increase awareness of microspherophakia as a possible ocular association of Marfanoid habitus. 

## Case presentation

**Figure 1:**
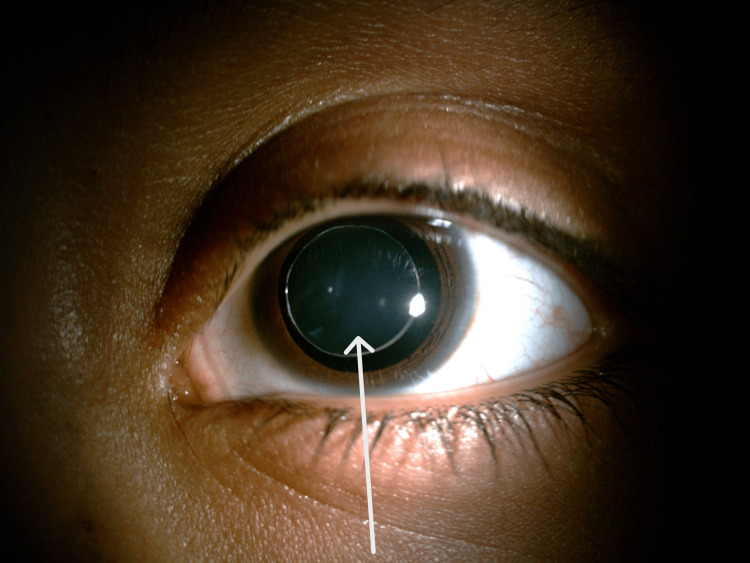
Left eye microspherophakia visible on diffuse illumination.

**Figure 2:**
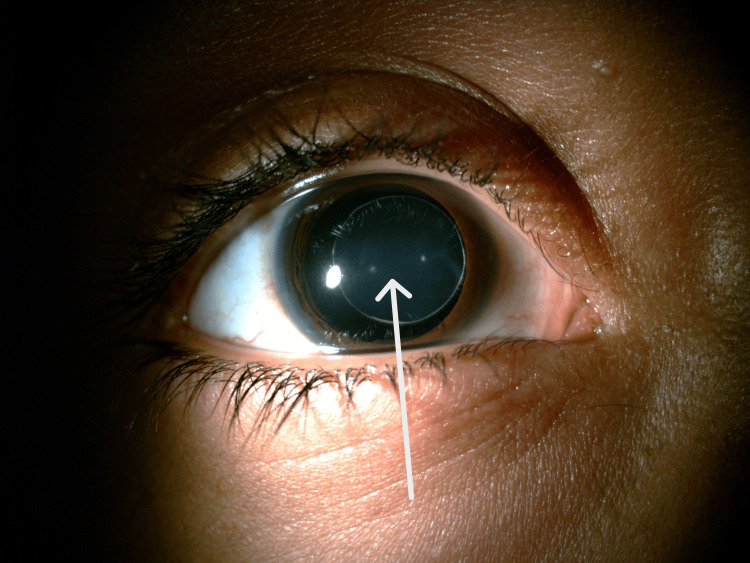
Right eye microspherophakia visible on diffuse illumination.

**Figure 3:**
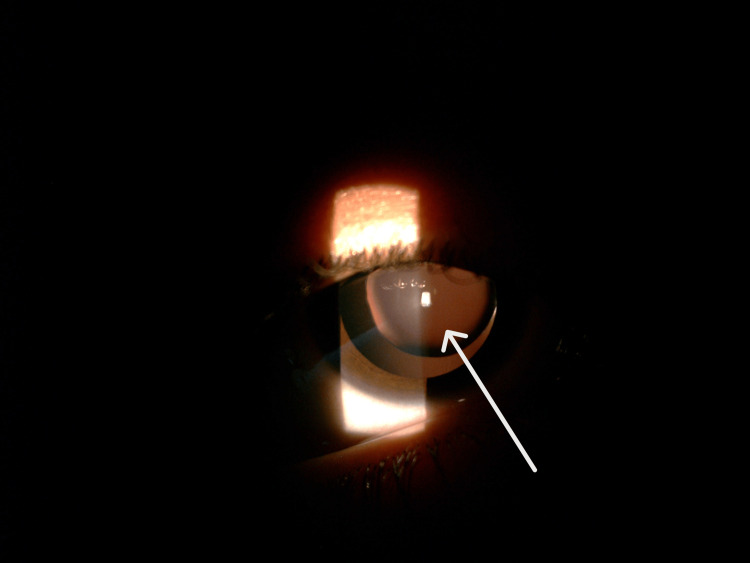
Right eye microspherophakia on retroillumination.

A 12-year-old female child presented to our Paediatric Ophthalmology Department with complaints of painless progressive loss of vision in both eyes since the age of five years. Her past medical and surgical history was unremarkable. Her family history was negative for any similar complaints. She was using glasses since the age of six years and frequently changed her glasses on six monthly eye exams. On examination, she had unaided visual acuity (VA) of counting fingers (CF) at 4 meters for both eyes; best corrected to 6/9 with -10.00 diopter sphere (DS) for both eyes. The patient's dilated anterior segment exam revealed bilateral anteriorly displaced microspherophakia as can be seen in Figure 1 which shows microspherophakia in left eye. Figure 2 shows microspherophakia in the right eye and Figure 3 shows microspherophakia on retroillumination. Her gonioscopic examination demonstrated a 360-degree open angle in both eyes. A healthy optic disc was confirmed through a posterior segment exam, and a healthy macula and retinal periphery were observed in both eyes. Her intraocular pressure (IOP) was 11 millimeters of Mercury (mmHg) in the right eye and 12 mmHg in the left eye. Her ultrasound brightness (USG B) scan of eyes detected no posterior staphyloma bilaterally. On investigations, the patient had an axial length of 27.5 mm and 27.7 mm in the right and left eye, respectively.

A thorough systemic exam of the patient was conducted. She displayed a tall and lean stature and an abnormally positioned mandible, indicative of retrognathia. She had an increased arm span-to-height ratio (height: 155 cm, arm span: 158 cm). Besides that, the systemic exam was unremarkable.

The patient was referred to the pediatrician and cardiologist to rule out valvular heart disease which revealed a normal examination and basic workup. She was advised to continue wearing her prescription eyeglasses. Patient and her mother were counselled in detail regarding the symptoms of angle closure glaucoma. Furthermore, she was scheduled for a three-monthly follow-up checkup for a detailed slit lamp exam and IOP check.

## Discussion

This case report highlights an extremely rare presentation of microspherophakia with an anteriorly displaced lens in a female child with Marfanoid habitus. Microspherophakia is a rare anomaly of the lens, characterized by refractive myopia and increased thickness of the lens. Ectopia lentis is a displacement of the lens from its normal position, which can be caused by several factors, including trauma and underlying systemic conditions such as Marfan syndrome [1].

Microspherophakia is associated with a very high incidence of certain complications. The existing literature tells us that lens subluxation occurs in more than 40% of patients with microspherophakia [3]. The occurrence of glaucoma in such cases may range from 44.4 to 51% of cases [2]. Multiple processes can lead to glaucoma in patients with microspherophakia. The spherical lens can advance due to a high anterior lens curvature and loosening of the zonular fibers, which causes iridolenticular contact and pupil block [10]. The other reported mechanisms include angle anomalies with hypoplasia of the angle structures, chronic pupillary block without full angle closure, and trabecular crowding by the spherophakic lens [3,10].

While there have been isolated case reports of microspherophakia associated with Marfan syndrome, bilateral anteriorly displaced microspherophakia is a rare presentation, and its association with Marfanoid habitus has not been reported in the literature to date [5]. The systemic and ocular features observed in our patient were consistent with Marfanoid habitus, including tall stature, retro-ganthia and increased arm span compared to body length [8]. These findings raise the possibility of an ocular association between microspherophakia and Marfanoid habitus.

## Conclusions

Bilateral anteriorly displaced microspherophakia with Marfanoid habitus is a rare clinical presentation and requires long-term follow-up owing to changing refractive error due to progressive change in the axial length of the eye. These patients and their parents need a detailed counselling re-emphasizing the requirement of routine follow-up due to the imminent risk of angle closure glaucoma and endothelial cell loss secondary to anterior subluxation of microspherophakic lens. A detailed evaluation by Pediatrician and Cardiologist is also mandatory on first visit to rule out systemic associations and a multidisciplinary approach is often required in the diagnosis and management of patients with Marfanoid habitus. Genetic studies where available can help in the definitive diagnosis and differentiation of Marfanoid habitius from Marfan syndrome.

## References

[1] Yu X, Chen W, Xu W (2020). Diagnosis and treatment of microspherophakia. J Cataract Refract Surg.

[2] Senthil S, Rao HL, Hoang NT, Jonnadula GB, Addepalli UK, Mandal AK, Garudadari CS (2014). Glaucoma in microspherophakia: presenting features and treatment outcomes. J Glaucoma.

[3] Muralidhar R, Ankush K, Vijayalakshmi P, George VP (2015). Visual outcome and incidence of glaucoma in patients with microspherophakia. Eye (Lond).

[4] Khokhar S, Pangtey MS, Sony P, Panda A (2003). Phacoemulsification in a case of microspherophakia. J Cataract Refract Surg.

[5] Guo H, Wu X, Cai K, Qiao Z (2015). Weill-Marchesani syndrome with advanced glaucoma and corneal endothelial dysfunction: a case report and literature review. BMC Ophthalmol.

[6] Şimşek T, Beyazyıldız E, Şimşek E, Öztürk F (2016). Isolated microspherophakia presenting with angle-closure glaucoma. Turk J Ophthalmol.

[7] Chan RT, Collin HB (2002). Microspherophakia. Clin Exp Optom.

[8] Wozniak-Mielczarek L, Osowicka M, Radtke-Lysek A (2022). How to distinguish Marfan syndrome from Marfanoid habitus in a physical examination-comparison of external features in patients with Marfan syndrome and Marfanoid habitus. Int J Environ Res Public Health.

[9] Mizuguchi T, Matsumoto N (2007). Recent progress in genetics of Marfan syndrome and Marfan-associated disorders. J Hum Genet.

[10] Willoughby CE, Wishart PK (2002). Lensectomy in the management of glaucoma in spherophakia. J Cataract Refract Surg.

